# Safety and effectiveness of high-power thulium laser enucleation of the prostate in patients with glands larger than 80 mL

**DOI:** 10.1186/s12894-019-0437-9

**Published:** 2019-01-21

**Authors:** Ching-Hsin Chang, Tzu-Ping Lin, Jung-Yao Huang

**Affiliations:** 1 Taipei Medical University Hospital, Taipei, Taiwan; 2 Institute of Microbiology and Immunology, National Yang-Ming University, Taipei, Taiwan; 3 Taipei Veterans General Hospital, Taipei, Taiwan; 4 Shutien Uro Clinic, Taipei, Taiwan

**Keywords:** Thulium laser, Enucleation, Large, Prostate, Bladder outlet obstruction, Open prostatectomy

## Abstract

**Background:**

The efficacy of thulium laser prostate enucleation (ThuLEP) for large prostates is unclear. This study aimed to explore the expanded utility of 150–200-W ThuLEP by studying patients with a large prostate (> 80 mL).

**Methods:**

We retrospectively reviewed records of 125 patients with large prostate glands (> 80 mL) who underwent ThuLEP performed by a single surgeon from June 2012 to June 2016. The ThuLEP data from our previous pilot study were used as the control. Operative variables, patient profiles, preoperative and postoperative urine flow rates, prostate volume, and the international prostate symptom score (IPSS) were recorded and analyzed using Student’s *t*-test, the *z*-test, and logistic regression analysis.

**Results:**

Of 366 patients who underwent ThuLEP, 125 (34.15%) were enrolled. The ages and estimated prostate volumes were compared with those of the control. Overall, 39.2% underwent Foley placement and 4% received an anticoagulant agent preoperatively. Maximum urinary flow rates before and 3 and 12 months postoperatively were 9.93, 23.20, and 19.00 mL/s, respectively, which were generally equal to those of the control groups (*P* = 0.68, 0.18, 0.98, respectively). Preoperative and postoperative IPSSs were 27.09 and 7.35, respectively. The postoperative prostate-specific antigen was reduced by 85.59% in comparison to the preoperative level. The estimated prostate size was reduced by 74.17% postoperatively. The modified Clavien-Dindo classification system was used to identify the overall rate of complications, which was approximately 22.4%. The mortality rate was 0.8%.

**Conclusions:**

High-power ThuLEP is safe and effective for patients with large prostate glands (> 80 mL). Prostate enucleation using a high-power thulium laser is feasible for patients who exhibit contraindications for surgery.

## Background

Open prostatectomy (OP) offers the highest probability of symptomatic improvement and the lowest failure rate of all treatment modalities for bladder outlet obstruction (BOO) due to benign prostatic hyperplasia (BPH) [1]. Despite considerable blood loss and prolonged recovery, OP has been the traditional treatment option for extremely large prostates [2, 3]. The previous American Urological Association (AUA) and European Urological Association (EAU) guidelines (in 2001 and 2003) suggest that OP should be the treatment of choice for patients with prostate volumes of 80–100 mL [4, 5].

Holmium laser enucleation of the prostate (HoLEP) has been adopted by many urologists worldwide because of the increased commercial availability (beginning in 1994) of high-powered holmium:yttrium-aluminum-garnet lasers. Since then, numerous single-center and multicenter large-scale series and randomized clinical trials have demonstrated its effectiveness and safety when used to treat BOO [6, 7]. HoLEP has been proven to be an endourological alternative to OP for large prostates [8]. Furthermore, beginning in 2013, HoLEP became the new “gold standard” because it was associated with a low operative blood loss, recatheterization rate, and reintervention rate, shorter hospital stay, and other benefits [9]. The section “Laser treatments of the prostate” of the last EAU Male LUTS Guidelines (the 2018 edition) was adapted [10]. OP or endoscopic enucleation of the prostate such as holmium laser or bipolar enucleation of the prostate are the first choice of surgical treatment in men with a substantially enlarged prostate and moderate-to-severe LUTS. This treatment was strongly recommended for men with prostate size > 80 mL.

With the evolution of technology, thulium laser prostate enucleation (ThuLEP) has become a novel treatment option for BOO [11]. In 2005, the thulium laser entered clinical practice with a power of approximately 50–120 W, thereby offering advanced vaporization and hemostatic features [12, 13]. Thulium appears to provide improved vaporization ability, thereby ensuring smooth tissue incisions compared to those of the holmium laser. Holmiun laser can only be used in pulsed form; the thulium laser is continuous but pulsatile. This nature is enabling the surgeon to accurately remove the adenoma at the level of the surgical capsule because it is easier to distinguish the adenoma in this plane. Theoretically, regardless of the size of the prostate, ThuLEP can effectively clear the transition zone [11, 14].

The power of an advanced thulium device is 150–200 W. In our pilot study published in 2015, a high-power thulium laser was used to perform ThuLEP in patients from the general or unselected public. One year of follow-up showed that ThuLEP and traditional transurethral resection of the prostate (TURP) effectively alleviated subjective and objective voiding symptoms with a low rate of complications [15]. Limited evidence described favorable outcomes with ThuLEP that were comparable with those with OP, TURP, and HoLEP for prostates < 80 mL [16, 17]. The studies by Pearce at el and Gross et al. evaluated patients with prostates > 80 mL [18, 19]. The former study included only 25 patients (> 75 mL) [18], and these patients became one of the subgroups (24%, 266 in 1080 patients) in the latter study but were briefly discussed [19].

Limited number of randomized controlled trials with long-term follow-up support the efficacy of ThuLEP, there is a need for ongoing investigation of the technique [10]. Furthermore the efficacy of ThuLEP for large prostates is unclear.

To confirm ThuLEP as an endourological alternative to OP and HoLEP for numerous specific conditions, the application of ThuLEP for treating large prostates should be evaluated. Therefore, in this study, we assessed the outcomes (i.e., safety, feasibility) of ThuLEP for patients with a large prostate (> 80 mL).

## Methods

We retrospectively reviewed data from 125 patients who underwent ThuLEP performed by a single surgeon (JYH) between June 2012 and June 2016. Approval was received from the institutional review board (VGHIRB 2016–03-007 AC), along with a waiver for informed consent.

**Fig. 1:**
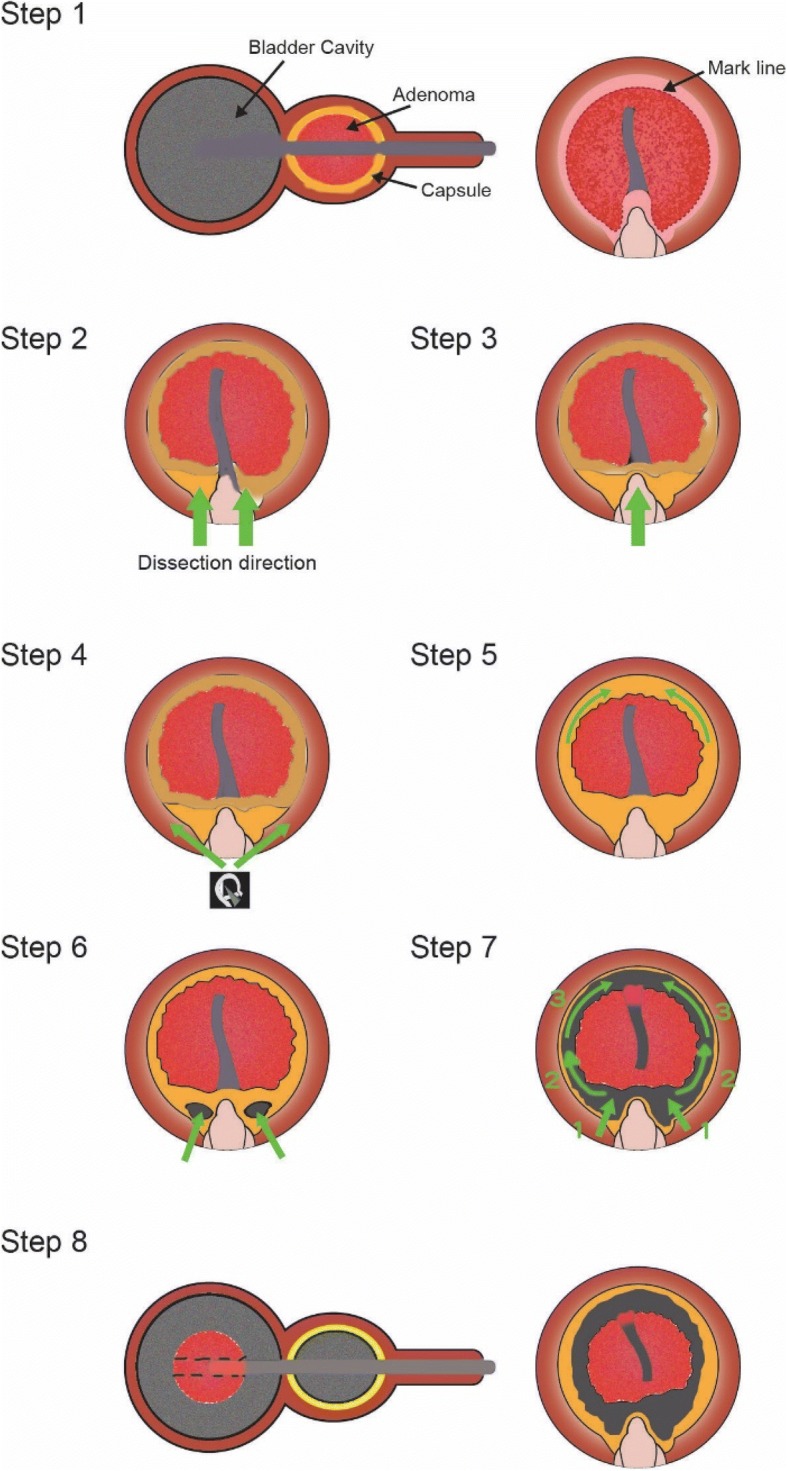
Illustration of the thulium laser prostate enucleation (ThuLEP) surgical technique. Step 1: Creation of the working space: coagulate the mucosa along the proximal side of the sphincter, sparing the verumontanum, and create the marked line. Step 2: Cut and extend from the marked line to the capsule in the 5 o’clock and 7 o’clock directions. The marked line that is proximal to the verumontanum is spared (in the 6 o’clock direction). Step 3: Extend from the cutting edge in the 5 o’clock and 7 o’clock directions to the 6 o’clock direction. Step 4: Place the guiding tube on the scope sheath and keep dissecting from the 6 o’clock direction to the opposite side (in the 5 to 4 and 3 o’clock directions and in the 7 to 8 and 9 o’clock directions, respectively). Step 5: The tissue in the 12 o’clock direction is relatively thin. Be careful to not cut too deep or shallow while cutting in this direction. Step 6: Cut along the prostate capsule and make a window to the urinary bladder space in the 5 and 7 o’clock directions. Step 7: Extend the cutting edge from the 5 and 7 o’clock directions to the 6 o’clock direction. Extend the edge along the lateral wall in the 12 o’clock direction. Step 8: Push the adenoma tissue into the bladder space, check the bleeder, and then perform the morcellation procedure

All patients, regardless of the presence of malignancy, had a prostate gland that was larger than 80 mL in volume, as measured using transabdominal ultrasonography and the prolate ellipsoid volume formula. No exclusion criteria were applied during chart review. The Cyber 150 device (Quanta System, Solbiate Olona, Varese, Italy) was used with 150–200 W of energy in continuous mode (Fig. 1). The Piranha Morcellator device (Richard Wolf, Knittlingen, Germany) was used after the enucleation step. Postoperative Foley catheter irrigation was performed, and then the catheter was removed the day after surgery. Patients who underwent ThuLEP during our pilot study were considered as a control group [15]. Data regarding surgical variables, patient status, preoperative and postoperative urinary flow rates, prostate volume, and the international prostate symptom score (IPSS) were extracted from each patient’s medical records. Follow-up consisted of written questionnaires that were sent in June 2017 and telephone interviews.

The database from published articles were introduced as the the OP, HoLEP, and ThuLEP control group [15, 20, 21]. Data were analyzed using Student’s *t*-test, the *z*-test, and logistic regression analysis with MedCalc software (Medcalc, Mariakerke, Belgium). For all statistical comparisons, significance was assumed when *P* <  0.05.

## Results

Of 366 patients (with all size of prostate glands) who underwent ThuLEP during a 4-year period, 125 (34.15%, with glands > 80 mL) were enrolled. Fifteen patients exhibited malignant pathological findings (Table 1). Compared to the ThuLEP group in the pilot study, patients in this study were younger (mean ± standard deviation, 71.85 ± 8.89 years; *P* = 0.02) and had a higher estimated prostate volume (106.80 ± 45.77 mL, range 80–332; *P* <  0.001) [15]. Additionally, patients in our study were generally older and had a larger prostate than did the general public. Of these patients, 39.2% underwent Foley placement, and 4% were treated with an anticoagulant agent preoperatively.

**Table 1:** Baseline characteristics and comparisons with the control groups

	Our study			Pilot study: ThuLEP [15]		Pilot study: TURP [15]	
	Total	Malignancy	Benign		*P*-value^**^		*P*-value^**^
Patients	125	15	110	29		30	
Age, years	71.85 ± 8.89	78.81 ± 7.17	71.07 ± 8.68	76.1 ± 9.4	0.02	72.6 ± 7.4	0.62
PSA level, ng/mL	18.40 ± 37.35	37.03 ± 60.34	16.14 ± 33.26	5.0 ± 5.4	0.49	8.3 ± 7.9	0.47
Anticoagulant agent use	5 (4%)	0	5 (4.45%)	15 (51.7%)		6 (20.0%)	
Estimated prostate volume, mL	106.80 ± 45.77	106.80 ± 70.76	106.40 ± 41.25	57.2 ± 25.1	< 0.01	64.7 ± 32.5	< 0.01
Preoperative Foley placement	49 (39.2%)	5 (100%)	44 (40%)	3 (10.34%)		7 (23.33%)	
Transfusion rate	4 (3.2%)	1 (6.67%)	3 (2.72%)	4 (13.79%)		8 (26.67%)	
Reduction ratio of the PSA level 3 months postoperatively (%)	85.59 ± 14.92			39.21 ± 44.95	< 0.0001	70.22 ± 28.86	0.0047
Reduction ratio of the estimated prostate size postoperatively (%)	74.17 ± 11.27			47.24 ± 16.20	< 0.0001	49.01 ± 17.79	< 0.0001

PSA, prostate-specific antigen, ThuLEP, thulium laser enucleation of the prostate, TURP, transurethral resection of the prostate

**Compared with the total group using Student’s t-test

**Fig. 2:**
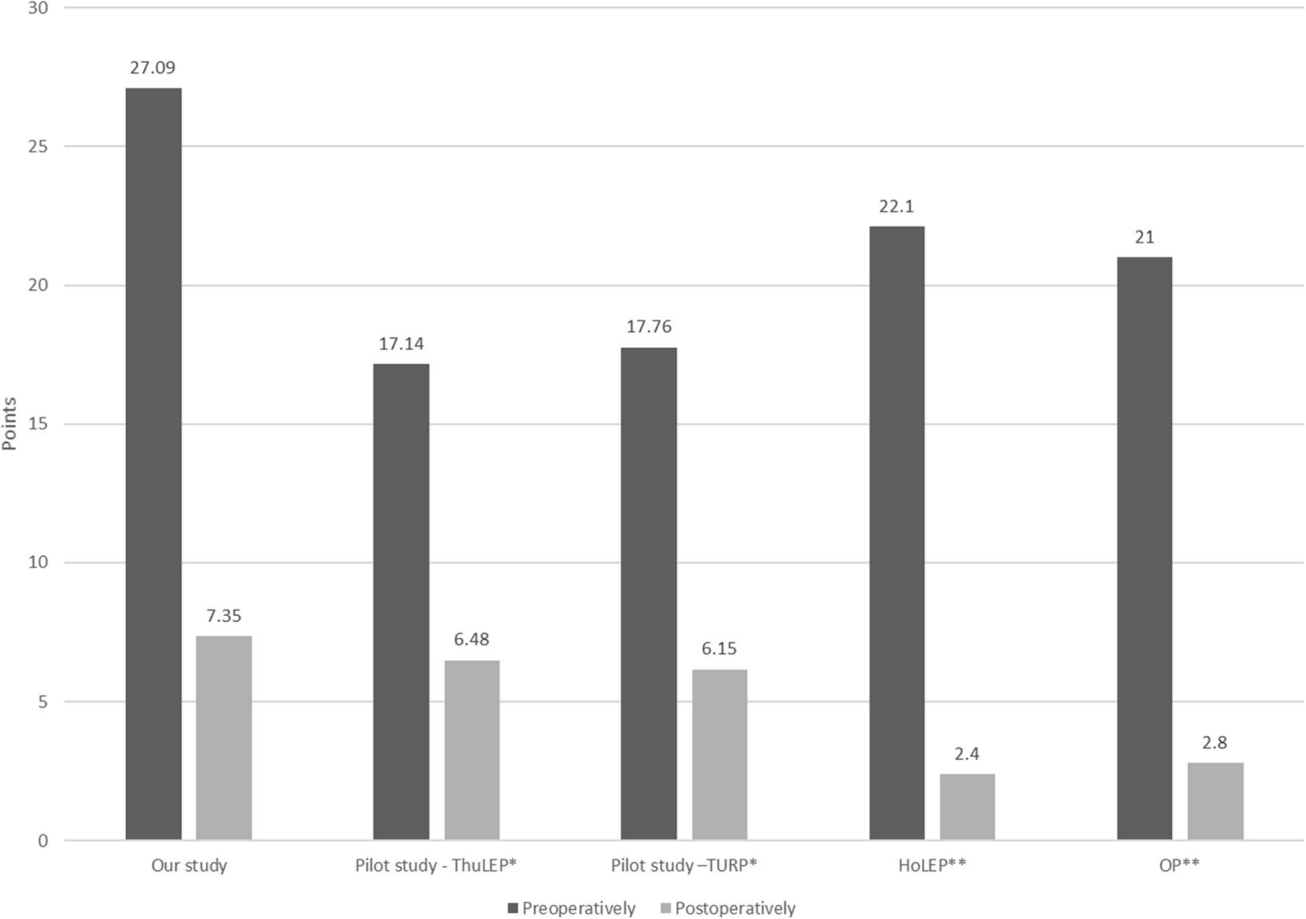
Preoperative and postoperative IPSSs are decreased dramatically in all studies. The IPSSs were measured preoperatively and postoperatively. IPSS, international prostate symptom score; ThuLEP, thulium laser prostate enucleation; HoLEP, holmium laser enucleation of the prostate

The preoperative IPSS was 27.09 ± 5.91, and the postoperative IPSS was 7.35 ± 5.89 in this study group. The control groups included the ThuLEP, HoLEP, and OP groups (Fig. 2) [15, 20]. These data indicated a higher preoperative IPSS and larger prostates in the study group, which were relieved after surgery, than in the control groups (*P* < 0.001, 0.51) (Table 2).

**Table 2:** Perioperative profiles and comparisons

		Our study: Total (*n* = 125)	Pilot study: ThuVEP [15] (*n* = 29)		Pilot study: TURP [15] (*n* = 30)	
				*P*-value ^*^		*P*-value^*^
IPSS, points	Preoperatively	27.09 ± 5.91	17.14 ± 5.08	< 0.01	17.76 ± 4.27	< 0.01
IPSS, points	Postoperatively	7.35 ± 5.89	6.48 ± 3.73	0.51	6.15 ± 4.43	0.49
PVR volume, mL	Preoperatively	329.76 ± 350.52	138.64 ± 127.74	0.01	90.91 ± 66.53	< 0.01
Maximum UFR, mL/s		9.93 ± 5.02	10.5 ± 4.86	0.68	10.84 ± 4.74	0.46
Mean UFR, mL/s		5.40 ± 2.18	5.02 ± 2.62	0.55	4.58 ± 2.15	0.13
PVR volume, mL	1 week postoperatively	60.67 ± 74.08				
Maximum UFR, mL/s		19.39 ± 12.01				
Mean UFR, mL/s		11.24 ± 6.38				
PVR volume, mL	1 month postoperatively	53.97 ± 36.05				
Maximum UFR, mL/s		17.92 ± 9.3				
Mean UFR, mL/s		11.09 ± 5.78				
PVR volume, mL	3 months postoperatively	38.67 ± 18.52	68.24 ± 37.54	< 0.01	69.50 ± 47.85	< 0.01
Maximum UFR, mL/s		23.20 ± 6.87	16.26 ± 7.72	0.18	22.77 ± 10.46	0.01
Mean UFR, mL/s		10.20 ± 3.77	7.50 ± 3.07	0.76	12.15 ± 6.20	0.04
PVR volume, mL	6 months postoperatively	48.22 ± 45.85				
Maximum UFR, mL/s		9.38 ± 13.98	16.53 ± 6.99	0.02	22.67 ± 10.40	0.06
Mean UFR, mL/s		18.28 ± 8.96	8.69 ± 3.62	0.60	11.51 ± 5.74	0.58
PVR volume, mL	12 months postoperatively	41.33 ± 42.02				
Maximum UFR, mL/s		19.00 ± 18.30	18.40 ± 7.13	0.98	23.34 ± 13.73	0.53
Mean UFR, mL/s		7.50 ± 4.71	9.68 ± 4.45	0.72	12.99 ± 8.38	0.34

The estimated prostate volume was calculated as the volume of the entire gland using the prolate elliptical formula (height × width × length × π/6). Overall, 10.4% of patients returned as outpatients and had uroflow data at more than 1 year postoperatively. IPSS, international prostate symptom score; PVR, postvoid residual volume; URF, urinary flow rate; ThuLEP, thulium laser enucleation of the prostate; TURP, transurethral resection of prostate

*Compared with the total group using Student’s t-test

**Fig. 3:**
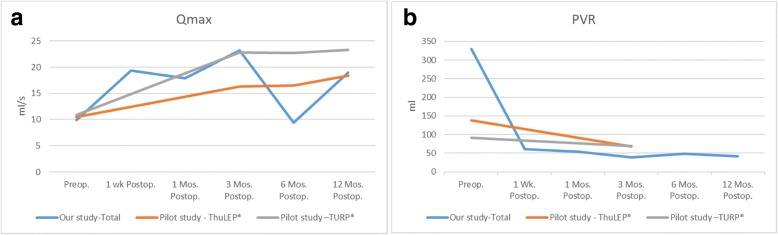
Perioperative Qmax (**a**) and PVR urine volume (**b**). The average of (**a**) the Qmax (mL/s) and (**b**) PVR urine volume (mL) from this study and our pilot study at different time points (preoperatively and postoperatively at 1 week, 1 month, 3 months, 6 months, and 12 months). Qmax, maximum flow rate; PVR, postvoiding residual; Wk., week; Mos., months; Preop., preoperatively; Postop., postoperatively; ThuLEP, thulium laser prostate enucleation; HoLEP, holmium laser enucleation of the prostate

The maximum urinary flow rates before and at 3 and 12 months postoperatively were 9.93 ± 5.02, 23.20 ± 6.87, and 19.00 ± 18.30 mL/s, respectively, which were generally equal to those found during the pilot study (*P* = 0.68, 0.18, and 0.98, respectively) (Fig. 3a, b). The postoperative prostate-specific antigen (PSA) was reduced by 85.59 ± 14.92% compared to the preoperative level. The estimated prostate size was reduced by 74.17 ± 11.27% postoperatively. These reduction rates were compatible with the characteristics of enucleation treatment.

The modified Clavien-Dindo classification system was used to identify complications following the pilot study [15, 22]. The overall complication rate was approximately 22.4%, whereas the mortality rate was 0.8% (Table 3). There were few differences between the OP, HoLEP, and ThuLEP control groups [20, 21], with only one case of mortality due to postoperative stroke 1 day later. The telephone follow-up time was 26.62 ± 13.76 months, and the completion rate of the postoperative IPSS questionnaire was 54.4%.

**Table 3:** Modified Clavien-Dindo classification system for reporting complications of prostate procedures

Grade	Modified Clavien-Dindo system for TURP	Our study		HoLEP [21]	OP [21]	ThuVEP [20]
		(*n*)	Incidence (%)			
1	Hematuria clot retention requiring bladder irrigation/clot evacuation/catheter traction	2	1.6	5	5	16.65
	Catheter block because of retained TUR chip	0	0	5	5	
	Failed trial without catheter with acute urinary retention requiring bedside recatheterization (CIC)	12	9.6			
2	Hemorrhage/hematuria requiring transfusion	4	3.2	0	13.3	4.34
	Urinary incontinence requiring a antimuscarinic agent	3	2.4			
3	Bladder neck contracture or urethral stricture, required surgical intervention	6	4.8	1.7	3.3	8.25
4	Acute myocardial infarction requiring admission to the ICU	0	0	0	1.7	0.79
	TUR syndrome requiring admission to ICU	0	0			
5	Death	1	0.8			0
Total		28	22.4	15	26.7	30.03

OP, open prostatectomy, CIC, clean intermittent catheterization, ICU, intensive care unit, HoLEP, holmium laser enucleation of the prostate, ThuLEP, thulium laser enucleation of the prostate, TURP, transurethral resection of prostate, TUR, transurethral resection

## Discussion

Urologists evaluate prospective BOO methods to ensure that they can be *extended* and *focused*. Such steps have been applied to all techniques that are commonly used today, such as OP, monopolar and bipolar TURP, photoselective vaporization techniques, and different laser therapies.

Extension of a technique involves its application across races and geographic regions. To achieve this, the learning curve for the technique should not be steep. The focus refers to its use in different groups, such as the elderly, patients with large glands, patients with heart disease, or those who use anticoagulants. After these two types of tests, the novel tool can be qualified and used in clinical practice.

Our pilot study demonstrated the ability of ThuLEP to be extended, and this study presented its ability to be focused [23]. To our knowledge, this is the first study to highlight 2 topics: the high-power thulium laser device and large prostates (> 80 mL). We used 80 mL as the cut-off value because that is the standard size of a large prostate according to the guidelines [4, 5]. This standard was applied to many endoscopic surgical procedures and different kinds of lasers (such as HoLEP) [9, 24]. Several previous studies discussed ThuLEP for patients with large prostates [25, 26]. However, their cut-off values varied (60 mL, 70 mL, and 100 mL) [27]. Our study was useful and crucial for determining the surgical management of patients with prostate volumes > 80 mL causing BOO secondary to BPH.

**Fig. 4:**
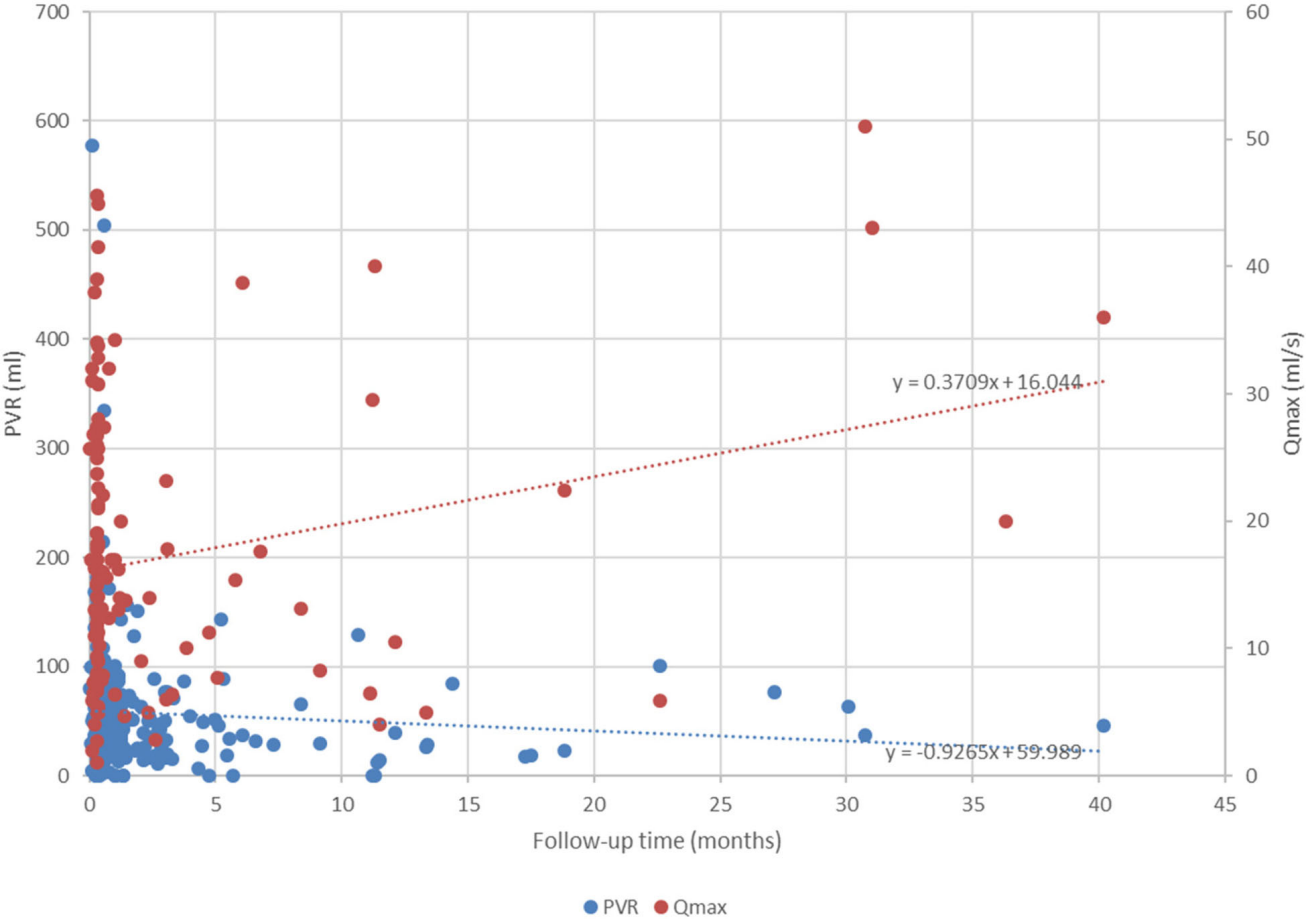
Perioperative PVR and Qmax. The PVR urine volume (mL, blue dots) and Qmax of uroflowmetry (mL/min, red dots) are shown based on the chart review. A regression line is drawn. PVR, postvoiding residual; Qmax, maximum urinary flow rate

When analyzing this study’s results, we attempted to ensure consistent demographic characteristics; therefore, we used control patient data from our pilot study. Following the case series analysis protocol, we compared several OP, HoLEP, and ThuLEP studies (Table 4) [20, 21]. Generally, our patients were younger, had bigger prostates, and exhibited more extensive urine retention. The reduction ratio of the estimated prostate size (%) in our study was 74.17 ± 11.27, which is comparable to the ratio reported by previous studies (82% for OP; 85% for HoLEP; and 61% for ThuVEP). The postvoiding residual (PVR) urine volume slope in our study was − 0.9265 (Fig. 4). However, in studies using OP, HoLEP, and ThuVEP, PVR urine volume slopes were − 13.411, − 9.64738, and − 41.1333, respectively (data not shown). These differences in PVR urine volumes and the incidence of complications may be due to differences in the collection method used. However, these incidence data were difficult to compare statistically because the patient population, surgeon, and technique were variable.

**Table 4:** Comparisons of perioperative profiles

		Our study: Total	HoLEP [21]		OP [21]		ThuVEP [20]	
				*P*-value*		*P*-value*		*P*-value*
Patients, N		125	60		60		1382	
Preoperative IPSS, points		27.09 ± 5.91	22.1 ± 3.3	< 0.0001	21.0 ± 3.6	< 0.0001	18.6 ± 7.6	< 0.0001
Postoperative IPSS, points		7.35 ± 5.89	2.4 ± 1.9	< 0.0001	2.8 ± 3.9	< 0.0001		
PVR volume, mL	Preoperatively	329.76 ± 350.52	280 ± 273	0.2915	292 ± 191	0.3438	156.3 ± 195.4	< 0.0001
Maximum UFR, mL/s		9.93 ± 5.02	3.8 ± 3.6	< 0.0001	3.6 ± 3.8	< 0.0001	10.7 ± 6.5	0.11
Mean UFR, mL/s		5.40 ± 2.18						
PVR volume, mL	1 week postoperatively	60.67 ± 74.08	10.2 ± 22.4	< 0.0001				
Maximum UFR, mL/s		19.39 ± 12.01	24.7 ± 3.3	< 0.0001				
Mean UFR, mL/s		11.24 ± 6.38						
PVR volume, mL	1 month postoperatively	53.97 ± 36.05	6.3 ± 18.7	< 0.0001	3.8 ± 8.7	< 0.0001		
Maximum UFR, mL/s		17.92 ± 9.3	26.9 ± 7.9	< 0.0001	26.6 ± 6.1	< 0.0001		
Mean UFR, mL/s		11.09 ± 5.78						
PVR volume, mL	3 months postoperatively	38.67 ± 18.52	7.2 ± 18.8	< 0.0001	3.0 ± 7.7	< 0.0001	32.9 ± 67.2	0.0186
Maximum UFR, mL/s		23.20 ± 6.87	27.6 ± 7.0	< 0.0001	27.3 ± 6.2	< 0.0001	19.2 ± 10.6	< 0.0001
Mean UFR, mL/s		10.20 ± 3.77						
PVR volume, mL	6 months postoperatively	48.22 ± 45.85	4.4 ± 11	< 0.0001	2.1 ± 6.0	< 0.0001		
Maximum UFR, mL/s		9.38 ± 13.98	29.9 ± 8.8	< 0.0001	27.0 ± 0.5	< 0.0001		
Mean UFR, mL/s		18.28 ± 8.96						
PVR volume, mL	12 months postoperatively	41.33 ± 42.02	5.8 ± 16.7	< 0.0001	6.4 ± 12.3	< 0.0001		
Maximum UFR, mL/s		19.00 ± 18.30	27.4 ± 9.7	< 0.0001	28.3 ± 7.5	< 0.0001		
Mean UFR, mL/s		7.50 ± 4.71						
Reduction ratio of the estimated prostate size postoperative (%)		74.17 ± 11.27	82		85		61	

IPSS, international prostate symptom score, Qmax, maximum urinary flow rate, OP, open prostatectomy, PVR, postvoid residual volume, UFR, urinary flow rate. *Comparison with our study using a two-tailed z-test

Our measured complication rate may be related to the large number of failed trials, as indicated by the increased number of patients who experienced urine retention postoperatively. These subgroups of complications were not considered in other studies (Table 3). Similarly, Gross et al. reported the complications of 266 patients with a large prostate [19]. The rates of acute urinary retention, re-operation, transfusion, and mortality were 7.5, 5.3, 2.6, and 0%, respectively. These rates seem to be slightly lower than those in our study (9.6, 4.8, 3.2, and 0.9%, respectively). The association between using of high-power Laser and the complications, especially the bladder neck stenosis, was not clear and required further evaluation. However, most importantly, only 1 case of mortality occurred in our study, which showed an uncertain relationship with the operation.

Randomized clinical trials are most suitable for testing the efficacy and safety of various methods. However, there have been only a few such studies. Although our study demonstrated the experience of real-world patients, the traceability of our patients was limited and impacted data collection. We sent questionnaires and conducted telephone interviews to avoid any error due to selection bias. The methods used to collect patient data in previous multicenter and national studies were unavailable, likely because they are time-consuming and resource-intensive [20].

There were a few limitations to our study. We encountered patients who elected to undergo the surgical procedure described in this study after seeking a second opinion at our clinic. For example, some patients had undergone Foley catheterization performed by their general physician or another urologist. The mean PSA level from the benign subgroup was 16.14  ± 33.26 ng/mL, which is very high compared to that reported in the literature before enucleation [16, 17]. However, the PSA density was equal to that in the pilot study. Thus, this result might reflect the presence of a larger prostate size or urinary retention with catheterization preoperatively.

Moreover, some patients were unable to undergo extensive follow-up at our clinic and returned to their general physicians. The high variation in follow-up (mean ± standard deviation, 26.62 ± 13.76 months) reflects this trend. Certain patients who were unhappy with the outcomes often visited the clinic more frequently and for a longer period. This may have affected our measurements of surgical outcomes (Fig. 3a, b). By excluding data of those who answered our questionnaires by mail or telephone, only 13 patients (10.4%) returned as outpatients and had uroflow data at more than 1 year postoperatively. Although our short-term postoperative outcomes appeared promising, further investigations of long-term efficacy are needed.

## Conclusions

The latest guideline and consensus demonstated OP or holmium laser or bipolar enucleation of the prostate are the first choice of surgical treatment in men with moderate-to-severe LUTS, and prostate size > 80 mL. Although OP and HoLEP have been described for large prostates, our study confirmed that ThuLEP is a suitable endourological alternative. High-power ThuLEP (i.e., approximately 150–200 W) is safe and effective for patients with large prostates (> 80 mL).

Therefore, promising outcomes associated with ThuLEP suggested that enucleation using a high-power laser is feasible for patients with large prostate > 80 mL.

## Abbreviations

AUA: American Urological Association
BOO: Bladder outlet obstruction
BPH: Benign prostatic hyperplasia
EAU: European Urological Association
HoLEP: Holmium laser enucleation of the prostate
IPSS: International prostate symptom score
OP: Open prostatectomy
PSA: Prostate-specific antigen
PVR: Postvoiding residual
ThuLEP: Thulium laser prostate enucleation.
TURP: Transurethral resection of the prostate
